# Alteration in Oral Microbiome Among Men Who Have Sex With Men With Acute and Chronic HIV Infection on Antiretroviral Therapy

**DOI:** 10.3389/fcimb.2021.695515

**Published:** 2021-07-14

**Authors:** Shuang Li, Junping Zhu, Bin Su, Huanhuan Wei, Fei Chen, Hongshan Liu, Jiaqi Wei, Xiaodong Yang, Qiuyue Zhang, Wei Xia, Hao Wu, Qiushui He, Tong Zhang

**Affiliations:** ^1^ Beijing Key Laboratory for HIV/AIDS Research, Center for Infectious Diseases, Beijing Youan Hospital, Capital Medical University, Beijing, China; ^2^ Department of Medical Microbiology, Capital Medical University, Beijing, China; ^3^ Institute of Biomedicine, Research Center for Infections and Immunity, University of Turku, Turku, Finland

**Keywords:** human immunodeficiency virus, oral microbiome, 16S rRNA sequencing, antiretroviral therapy, men who have sex with men

## Abstract

Despite the antiretroviral therapy (ART), human immunodeficiency virus (HIV)-related oral disease remains a common problem for people living with HIV (PLWH). Evidence suggests that impairment of immune function in HIV infection might lead to the conversion of commensal bacteria to microorganisms with increased pathogenicity. However, limited information is available about alteration in oral microbiome in PLWH on ART. We performed a longitudinal comparative study on men who have sex with men (MSM) with acute HIV infection (n=15), MSM with chronic HIV infection (n=15), and HIV-uninfected MSM controls (n=15). Throat swabs were collected when these subjects were recruited (W0) and 12 weeks after ART treatment (W12) from the patients. Genomic DNAs were extracted and 16S rRNA gene sequencing was performed. Microbiome diversity was significantly decreased in patients with acute and chronic HIV infections compared with those in controls at the sampling time of W0 and the significant difference remained at W12. An increased abundance of *unidentified Prevotellaceae* was found in patients with acute and chronic HIV infections. Moreover, increased abundances of *Prevotella* in subjects with acute HIV infection and *Streptococcus* in subjects with chronic HIV infection were observed. In contrast, greater abundance in *Lactobacillus*, *Rothia*, *Lautropia*, and *Bacteroides* was found in controls. After effective ART, *Bradyrhizobium* was enriched in both acute and chronic HIV infections, whereas in controls, *Lactobacillus*, *Rothia*, *Clostridia*, *Actinobacteria*, and *Ruminococcaceae* were enriched. In addition, we found that lower CD4^+^ T-cell counts (<200 cells/mm^3^) were associated with lower relative abundances of *Haemophilus*, *Actinomyces*, unidentified *Ruminococcaceae*, and *Rothia*. This study has shown alteration in oral microbiome resulting from HIV infection and ART. The results obtained warrant further studies in a large number of subjects with different ethnics. It might contribute to improved oral health in HIV-infected individuals.

## Introduction

Human immunodeficiency virus (HIV) infection is characterized by rapid and substantial loss of CD4^+^ T cells that impairs host defense and increases the risk of opportunistic microbial infections. Worldwide, there are approximately 38 million people living with HIV infection (PLWH), with about 25.4 million receiving antiretroviral therapy (ART). Although PLWH with ART might achieve a stable virus suppression, several oral diseases, such as oropharyngeal candidiasis (OPC), oral hairy leukoplakia, periodontitis, oral warts, ulcers, herpes, and Kaposi’s sarcoma, are frequently reported (Goldberg et al., 2015; Heron and Elahi, 2017; El Howati and Tappuni, 2018).

It has been shown that impairment of immune function in HIV infection might lead to the conversion of commensal bacteria to microorganisms with increased pathogenicity and contributes to opportunistic infections (Saxena et al., 2016; Griffen et al., 2019). Several studies reported the changes of gut microbiota composition observed in HIV infection (Sun et al., 2016; Zhou et al., 2018; Rocafort et al., 2019), including the increase in *Prevotella* and decrease in *Bacteroides* (Dillon et al., 2014; Mutlu et al., 2014). Alterations in the gut microbiota will eventually lead to an imbalance between microbes and their metabolites and could result in HIV-associated immune activation and inflammation (Dinh et al., 2015; Zevin et al., 2016). In addition, various studies have demonstrated that microbial translocation is a cause of HIV-associated immune activation and inflammation (Brenchley et al., 2006; Marchetti et al., 2013; Koay et al., 2018). Early in HIV infection, the observed loss of Th17 cells results in impaired integrity of the mucosal epithelial barriers, leading to microbial translocation from the gut lumen into the systemic circulation (Brenchley et al., 2006; Mudd and Brenchley, 2016). Although a great number of studies have focused on the contribution of gut microbiota to HIV infection, a handful of studies have also characterized the oral microbiome in HIV-infected individuals (Fulcher, 2020). Li et al reported that in comparison to the HIV-negative individuals, PLWH had higher levels of total cultivable microbes in saliva, including oral *streptococci*, *Streptococcus mutans*, *lactobacilli*, and *Candida* (Li et al., 2014). Recently Annavajhala et al. have shown that oral microbiome communities likely contribute to systemic inflammation and immune activation in PLWH (Annavajhala et al., 2020). However, data are still limited about alterations in oral microbiome in HIV infection on ART.

Previous studies have also demonstrated that the abundance and diversity of oral microbiomes in PLWH were significantly different from those observed in healthy controls. Indeed after ART, the oral microbiome composition was not completely recovered, although it turned to be similar to that observed in healthy controls (Presti et al., 2018; Li et al., 2020). It is known that the composition and homeostasis of oral microbiome are affected by multiple factors, such as diet, medication, and host responses (Samaranayake and Matsubara, 2017). It has been shown that *Prevotella*-rich microbiomes in the gut are associated with MSM and with HIV infection status (Noguera-Julian et al., 2016; Armstrong et al., 2018). Therefore, HIV-uninfected MSM could serve as useful controls when the effect of HIV infection itself on the oral microbiomes is studied. Further, to explore the impact of ART treatment on oral microbiomes, longitudinal studies and serial samples are needed. In this longitudinal study, we aimed to compare and identify changes in the oral microbiome in MSM with acute and chronic HIV infection before and after ART. Paired throat swab samples were collected within an interval of 12 weeks.

## Materials and Methods

### Study Subjects and Sample Collection

Participants with acute HIV infection or chronic HIV infection (both were from MSM population) were recruited from Beijing Youan Hospital, Beijing, China from May to November 2019. All acute HIV-infected individuals (referred as A0, n=15) and chronic HIV-infected individuals (B0, n=15) had not initiated ART. Fifteen HIV-uninfected MSM (D, n=15) were selected and included as controls. Throat swabs were collected from controls and all HIV-infected ART-naive individuals at the time of recruitment. Thereafter, all acute HIV-infected individuals (A12, n=15) and chronic HIV-infected individuals (B12, n=15) received ART, and throat swabs were collected at 12 weeks of ART. Acute HIV infection was defined as a positive HIV RNA but with negative or indeterminate HIV antibody results. Participants who have used antibiotics, probiotics, and prebiotics within the previous 4 weeks were excluded. In addition, the patients accompanied by active opportunistic infection and HBV/HCV were also excluded. The demographic and clinical characteristics of the study subjects are shown in **Table 1**. This study received approval from the ethics committee of the Beijing Youan Hospital ([2018]025), and all participants provided written informed consents.

**Table 1:** Demographic and clinical characteristics of the study subjects.

1		Acute HIV infection (n = 15)	Chronic HIV infection (n = 15)	Controls (n = 15)
Age (years, IQR)	29.4 (19–50)		37.3 (23–55)	40.1 (26–55)
Nadir CD4^+^ T-cell count (cells/mm^3^, IQR)	387 (293.8–531)		376.2 (303.8–532)	—
CD4^+^ T-cell count (cells/mm^3^, IQR)	397.4 (259–562.7)		486.5 (402–543)	—
Viral load (copies/ml)	56407 (7,156–97,871)		36592 (6,112–39,112)	—

All study subjects were male and had sex with men. IQR indicates interquartile range.

### 16S rRNA Gene Sequence Analysis

Altogether 73 throat swabs from PLWH (except two patients at 12 weeks of ART were lost to follow-up), and controls were collected. The clean throat swabs were used by the clinical nurses to collect oral samples from the posterior throat and tonsil areas. The swabs were used to repeatedly wipe the sampling site two times and quickly transported and stored at −20°C in the laboratory before polymerase chain reaction (PCR). Genomic DNAs were extracted from swabs using the QIAamp Fast DNA Stool Mini Kit (50) and amplified by PCR for the sequencing of 16S rRNA V4-V5 region. Sequencing libraries were generated using Illumina TruSeq DNA PCR-Free Library Preparation Kit (Illumina, USA). The library quality was assessed on the Qubit@ 2.0 Fluorometer (Thermo Scientific) and Agilent Bioanalyzer 2100 system. At last, the library was sequenced on an Illumina NovaSeq platform and 250 bp paired-end reads were generated.

Paired-end reads were assigned to samples based on the unique barcodes and merged by using FLASH (V1.2.7). The raw tags were obtained, and the quality filtering was performed under specific filtering conditions to obtain the high-quality clean tags according to the QIIME (V1.9.1, http://qiime.org/scripts/split_libraries_fastq.html) quality control process. Then the tags were compared with the reference database (Silva database, https://www.arb-silva.de/) using UCHIME algorithm (UCHIME algorithm, http://www.drive5.com/usearch/manual/uchime_algo.html) to detect chimera sequences. The chimera sequences were removed, and the effective tags were finally obtained. Operational taxonomic units (OTUs) were clustered by Uparse software (Uparse v7.0.1001), and representative sequence for each OTU was screened for further annotation. For each representative sequence, the Silva Database was used based on Mothur algorithm to annotate taxonomic information. Alpha diversity is applied in analyzing complexity of species diversity for a sample, including Observed-species, Chao1, Shannon, Simpson, ACE, Good-coverage, and PD whole tree. All these indices in our samples were calculated with QIIME (Version 1.7.0) and displayed with R software (Version 2.15.3). Beta diversity analysis was used to evaluate differences of samples in species complexity. Beta diversity on both weighted and unweighted unifrac was calculated by QIIME software (Version 1.9.1). Principal Coordinate Analysis (PCoA) analysis was displayed by WGCNA package, stat packages, and ggplot2 package in R software (Version 2.15.3).

### Statistical Analysis

Alpha diversity and beta diversity in oral microbiome among groups were tested by the Wilcoxon rank-sum test. To assess the differences in the microbial abundance between samples, significance test was conducted with some statistical analysis methods, including *t*-test and LEfSe. Mann-Whitney test and Kruskal Wallis test were used for comparing continuous variables. Two sides of *p* < 0.05 were considered statistically significant.

## Results

### Study Population

Samples were collected from 15 patients with acute HIV infection, 15 patients with chronic HIV infection, and 15 healthy controls. All participants (≥18 years old) were MSM, and all PLWH with acute and chronic HIV infection had not initiated ART. Throat swabs were collected from controls and HIV-infected patients at baseline and 12 weeks of ART. The baseline CD4^+^ T-cell count was 387 cells/mm^3^ in acute HIV infection and 376.2 cells/mm^3^ in chronic HIV infection. After ART for 12 weeks, all patients had increased CD4^+^ T-cell counts, with 397.4 cells/mm^3^ in acute HIV infection and 486.5 cells/mm^3^ in chronic HIV infection (**Table 1**).

### Oral Microbiome Diversity in Study Subjects

**Figure 1:**
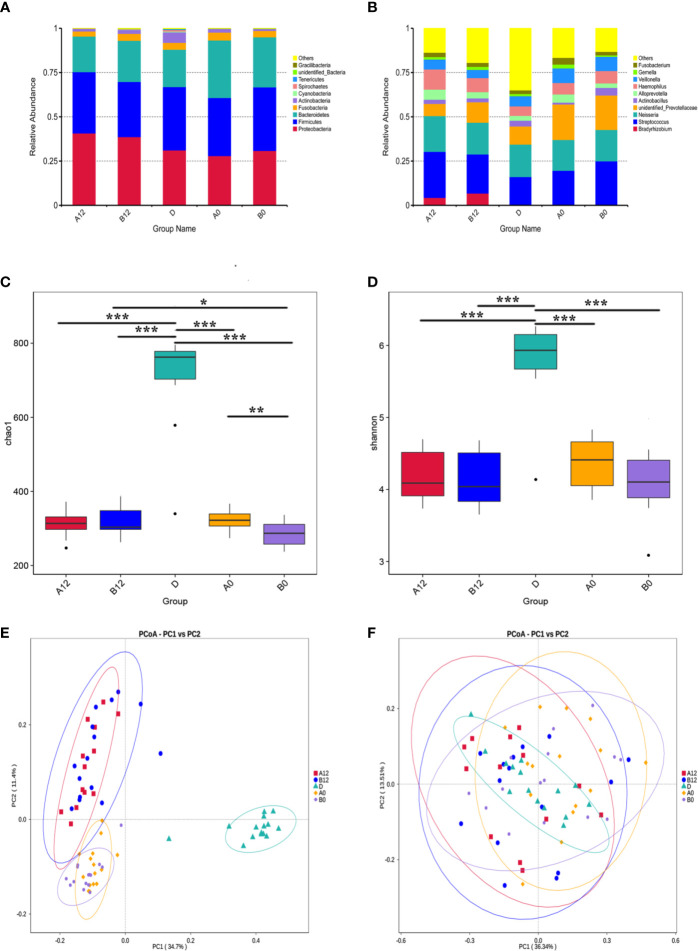
The composition, relative abundance, and diversity of oral microbiota in HIV-infected groups and controls **(A)** at the phylum level; **(B)** at the genus level; **(C)** Alpha diversity exemplified by the Chao1 index; **(D)** Alpha diversity exemplified by the Shannon index; **(E)** Beta diversity represented by Principal coordinate analysis (PcoA) of unweighted UniFrac distances; **(F)** Beta diversity represented by Principal coordinate analysis (PcoA) of weighted UniFrac distances. A0, people living with acute HIV infection at baseline; B0, people living with chronic HIV infection at baseline; D, HIV-uninfected controls; A12, people living with acute HIV infection after 12 weeks of ART; B12, people living with chronic HIV infection after 12 weeks of ART. *p < 0.05; **p < 0.01; ***p < 0.001.

Sequencing resulted in an average of 59,794 high-quality sequences after quality checks. Rarefaction curves and rank abundance showed a great sequencing depth and an even species distribution of the samples in our study (**Supplementary Figures S1A, B**). By clustering the sequences into OTUs with 97% similarity, an average of 683 OTUs were identified in controls. The corresponding numbers were 298 and 276 OTUs in the A0 and B0 groups, respectively. Following 12 weeks of ART, the corresponding numbers were 299 and 300 OTUs in the A12 and B12 groups. The following dominant phyla were observed in the oral microbiome: *Proteobacteria*, *Firmicutes*, *Bacteroidetes*, *Fusobacteria*, *Actinobacteria*, and *Spirochaetes*, *etc* (**Figure 1A**). These six dominant bacterial phyla accounted for more than 98% of the total oral microbiome. The most abundantly detected bacterial genus in the oral microbiome were *Streptococcus*, *Neisseria*, *unidentified Prevotellaceae*, with lower relative abundance of *Veillonella*, *Haemophilus*, *Actinobacillus*, *Alloprevotella*, and *Fusobacterium* in controls and the HIV-infected patients prior to ART. In addition, the abundance of *Bradyrhizobium* in both acute and chronic HIV infection groups was increased following 12 weeks of ART (**Figure 1B**).

We next used richness estimators (Chao) and diversity index (Shannon index) to compare microbial alpha diversity among different groups of samples. Compared with the control group D, the Chao1 index and Shannon index of oral microbiomes in both A0 group and B0 group were significantly decreased (**Figures 1C, D** **)** (all *p*<0.001). Although after 12 weeks of ART, the Chao1 index and Shannon index were still significantly lower in acute and chronic HIV-treated groups when compared with healthy controls (**Figures 1C, D** **)** (**Supplementary Table S1**) (all *p*<0.001). However, the Chao1 index of oral microbiomes in B12 group was significantly increased compared to B0 group (**Figure 1C**) (*p*<0.001). In addition, the Chao1 index in B0 group was significant decreased compared to A0 group (*p*<0.01), whereas the differences was not significant between the A12 and B12 (**Figure 1C**) (*p*=0.75). To identify the differences in microbial community composition of participants in these groups, the Principal Co-ordinates Analysis (PCoA) was used for beta diversity analysis. A conspicuous separation between HIV infection groups and control group was observed, beta diversity of oral microbiomes was significantly different between HIV-infected individuals and controls (**Figures 1E, F** **)** (*p*<0.001). There was no significant difference in beta diversity represented by PcoA of weighted UniFrac distances when comparing A0 group and B0 group (*p*=0.58), but there was a significant difference between groups A12 and B12 (*p*<0.001) **(** **Figure 1F** **)**. Likewise, no significant difference in beta diversity of weighted UniFrac distances was observed between A0 and A12 groups (*p*=0.32), but there was a significant difference between groups B0 and B12 (**Figure 1F**) (*p*<0.001).

### Oral Microbiome Dysbiosis in People Living With Acute and Chronic HIV Infection

**Figure 2:**
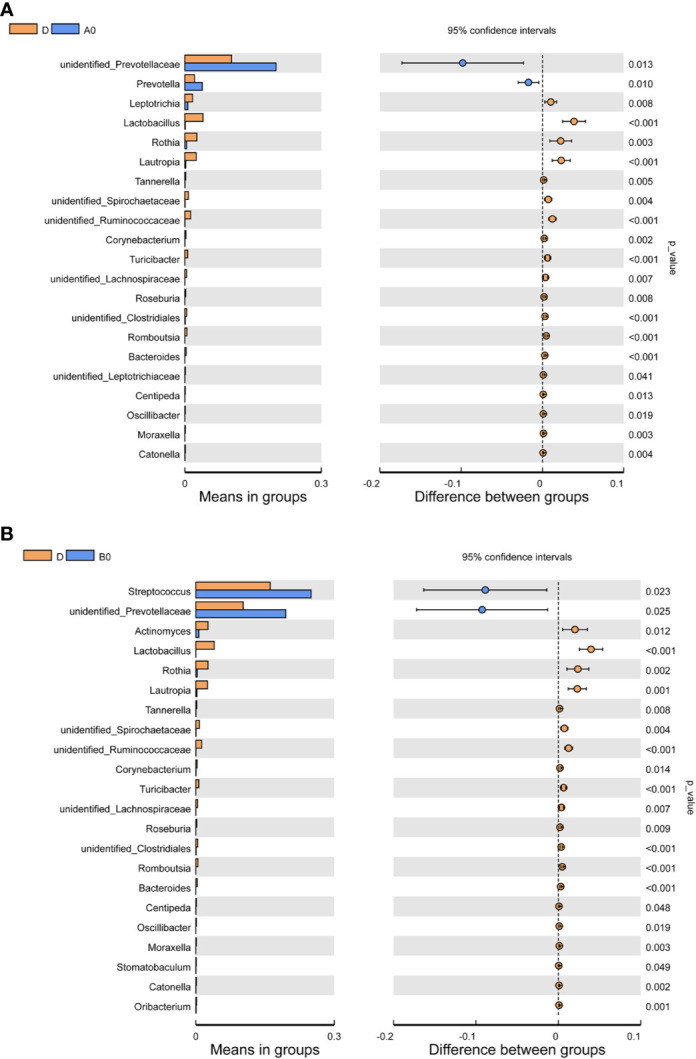
Comparisons of the relative abundance in oral microbiota at the genus level **(A)** Between A0 and D groups; **(B)** Between B0 and D groups. A0, people living with acute HIV infection at baseline; B0, people living with chronic HIV infection at baseline; D, HIV-uninfected controls; A12, people living with acute HIV infection after 12 weeks of ART; B12, people living with chronic HIV infection after 12 weeks of ART.

Differences in the composition of oral microbiome between controls and PLWH were analyzed. At the genus level, we observed the increased abundance of *unidentified Prevotellaceae* in both A0 and B0 groups. The abundances of *Prevotella* in the A0 group and *Streptococcus* in the B0 group were significantly increased. In contrast, several groups of bacteria, such as *Lactobacillus*, *Rothia*, *Lautropia*, and *Bacteroides* were found in greater abundance in D group (**Figures 2A, B** **)**.

### Effects of ART on the Oral Microbiome

**Figure 3:**
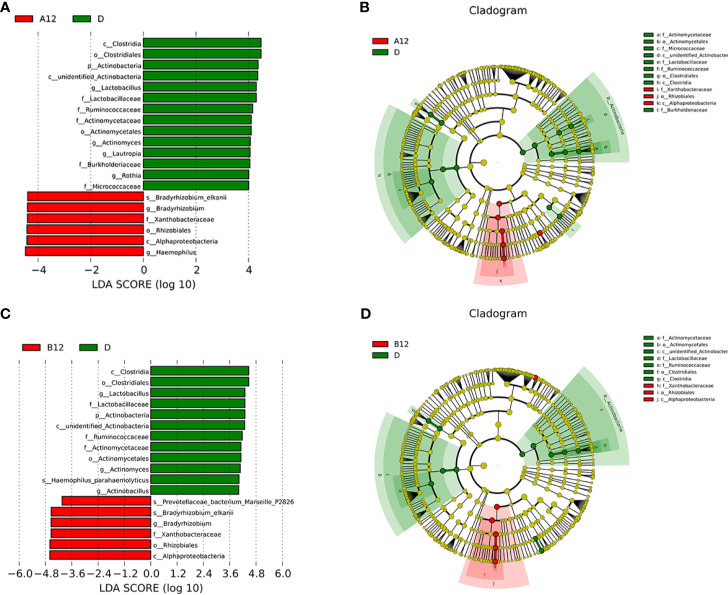
Linear discriminative analysis (LDA) effect size (LefSe) at the genus level. **(A, B)** shown between A12 and D groups and **(C, D)** between B12 and D groups. In **(A, B)**, LDA scores for the significant taxa in D group are represented on the positive scale (green), and LDA-negative scores represent enriched taxa in A12 group (red); and in **(C, D)**, LDA scores for the significant taxa in D group are represented on the positive scale (green), and LDA-negative scores represent enriched taxa in B12 group (red). A12, people living with acute HIV infection after 12 weeks of ART; B12, people living with chronic HIV infection after 12 weeks of ART; D, HIV-uninfected controls.

All of the PLWH included in this study received ART after the swab samples at baseline were taken. The second swabs were taken after 12 weeks of ART. LEfSe analyses showed that *Bradyrhizobium* was enriched in both A12 and B12 groups, whereas *Clostridia*, *Actinobacteria*, *Lactobacillus*, *Ruminococcaceae*, *Rothia* were enriched in the D group **(** **Figures 3A–D** **)**.

**Figure 4:**
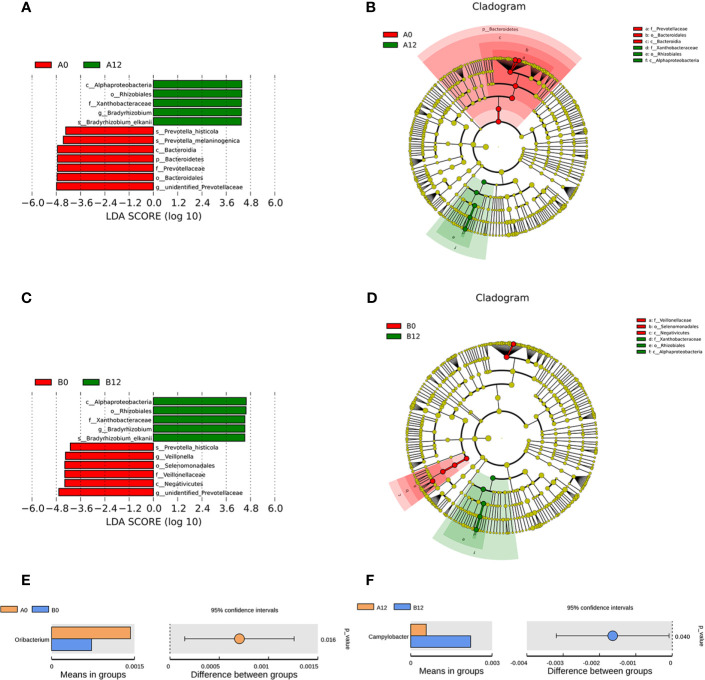
Linear discriminative analysis (LDA) effect size (LefSe) at the genus level. **(A, B)** shown between A0 and A12 groups and **(C, D)** between B0 and B12 groups. In **(A, B)**, LDA scores for the significant taxa in A12 group are represented on the positive scale (green), and LDA-negative scores represent enriched taxa in A0 group (red); and in **(C, D)**, LDA scores for the significant taxa in B12 group are represented on the positive scale (green), and LDA-negative scores represent enriched taxa in B0 group (red). Comparisons of the relative abundance in oral microbiota at the genus level **(E)** Between A0 and B0 groups; **(F)** Between A12 and B12 groups. A0, people living with acute HIV infection at baseline; B0, people living with chronic HIV infection at baseline; D, HIV-uninfected controls; A12, people living with acute HIV infection after 12 weeks of ART; B12, people living with chronic HIV infection after 12 weeks of ART.

Moreover, *Prevotella histicola*, *Prevotella melaninogenica*, *Bacteroidales*, and *unidentified Prevotellaceae* were enriched in the A0 group, while *Bradyrhizobium* were enriched in the A12 group **(** **Figures 4A, B** **)**. In the chronic HIV-infected patients, *Prevotella histicola*, *Prevotella melaninogenica*, *Veillonellaceae*, and *unidentified Prevotellaceae* were enriched in the B0 group, whereas the relative abundance of *Bradyrhizobium* in the B12 group was significantly higher **(** **Figures 4C, D** **)**.

Additionally, we also compared the composition of oral microbiome in people living with acute HIV infection and chronic HIV infection. Compared with A0 group, we noticed lower abundances of *Oribacterium* in the B0 group **(** **Figure 4E** **)**. However, after 12 weeks of ART, we found that the abundance of *Campylobacter* in the B12 group was significantly higher than those in the A12 group **(** **Figure 4F** **)**.

### Association of the Oral Microbiome With CD4^+^ T-Cell Count

**Figure 5:**
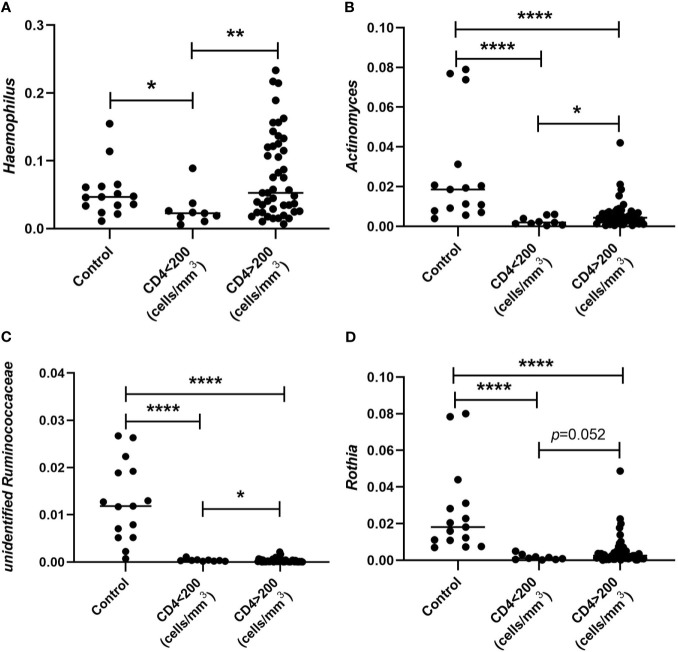
Comparison of relative abundances of *Haemophilus* **(A)**, *Actinomyces* **(B)**, *unidentified Ruminococcaceae* **(C)**, and *Rothia* **(D)** collected from subjects whose CD4^+^ T cells were defined < and CD4>200 cells/mm^3^ and HIV-uninfected MSM controls. *p < 0.05; **p < 0.01; ****p < 0.0001.

To study whether there is relationship between the alteration observed in oral microbiome and CD4^+^ T-cell count of patients, we performed analyses of these parameters. We found that the abundance of *Haemophilus* in patients with CD4<200 cells/mm^3^ was significantly decreased when comparing with HIV-uninfected controls and patients with CD4>200 cells/mm^3^. However, there was no significant difference between controls and patients with CD4>200 cells/mm^3^. In addition, the abundances of *Actinomyces*, *unidentified Ruminococcaceae*, and *Rothia* collected from both subjects with CD4 <200 cells/mm^3^ and subjects with CD4>200 cells/mm^3^ were significantly lower than those in HIV-uninfected subjects. Furthermore, lower CD4^+^ T-cell counts (<200 cells/mm^3^) were associated with lower relative abundances of *Haemophilus*, *Actinomyces*, *unidentified Ruminococcaceae*, and *Rothia* (**Figures 5A–D** **)**.

### Differences in Microbiota Metabolic Pathways Between HIV Infections and Healthy Controls

**Figure 6:**
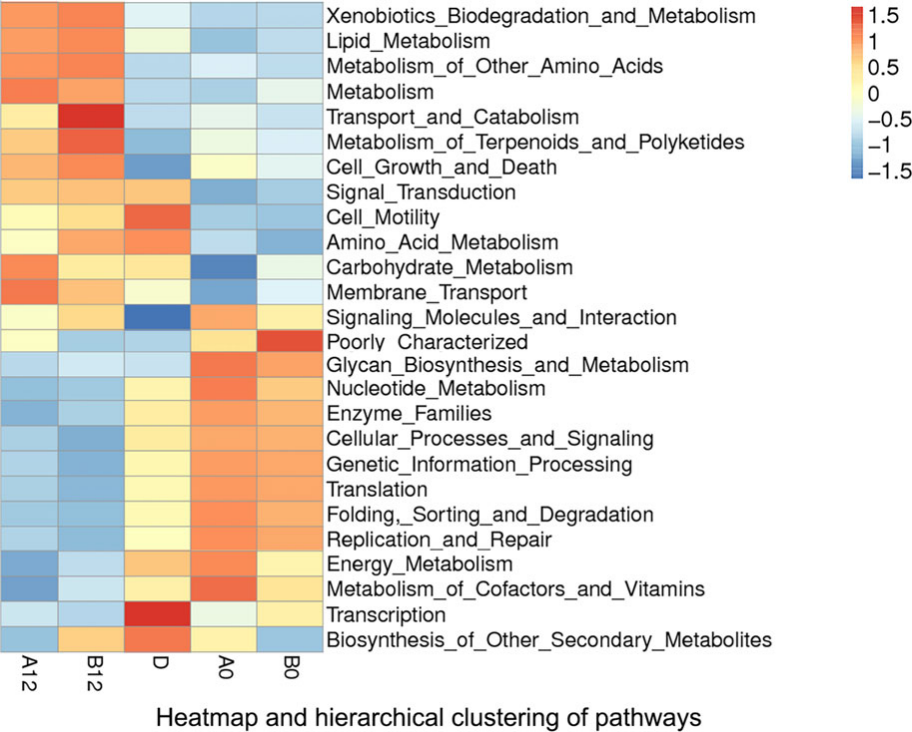
Heatmap and hierarchical clustering of pathways in A0, B0, D, A12 and B12 groups. A0, people living with acute HIV infection at baseline; B0, people living with chronic HIV infection at baseline; D, HIV-uninfected controls; A12, people living with acute HIV infection after 12 weeks of ART; B12, people living with chronic HIV infection after 12 weeks of ART.

According to the functional annotation, we used PICRUSt metagenome prediction to estimate the functional role of oral microbiome in PLWH and healthy controls. Compared with the D group, we noticed that pathways involved in cell growth and death, glycan biosynthesis, and metabolism increased in HIV-infected patients prior to ART (*p*<0.05). Moreover, replication and repair of DNA, genetic information processing, and translation-related pathways also increased in both A0 and B0 groups (*p*<0.05). In contrast, pathways related to xenobiotics biodegradation and metabolism, signal transduction, and cell motility (*p*<0.05) were decreased in both A0 and B0 groups (**Figure 6** and **Supplementary Figures S2A, B** **)**.

After 12 weeks of ART, comparison of pathways between HIV-infected groups and healthy controls showed that ART could partly reverse the changes of pathways in the acute and chronic HIV infection, but pathways related to metabolism, cell growth and death were still significantly increased (*p*<0.05), whereas transcription was significantly decreased in both A12 and B12 groups (*p*<0.05) **(** **Figure 6** and **Supplementary Figures S2C, D** **)**.

## Discussion

Our present study clearly showed that the OTUs identified in oral microbiome in people living with acute or chronic HIV infection were significantly lower compared with those in controls, and the number of OTUs in patients did not return to normal level even after 12 weeks of ART. Moreover, the alpha diversity of oral microbiomes in the patients was decreased significantly. In addition, beta diversity was also found to be different between patients and controls. Our results were in line with an earlier finding in which a decrease in microbial diversity in HIV-infected individuals was observed (Li et al., 2014). The decreased alpha diversity may be attributed to the increased proportions of opportunistic microbes as a result of immunocompromised state of patients (Li et al., 2014). Recently, Jiménez-Hernández et al. reported an increase of diversity parameters in salivary microbiota in HIV-infected individuals, mainly in those viremic ART-naive patients (Jimenez-Hernandez et al., 2019). It is conceivable that the impaired immune function resulting from HIV infection can disrupt the normally constituted commensal oral bacterial colonization, and the elevated viremia in untreated PLWH is associated with significantly higher proportions of potentially pathogenic *Veillonella*, *Prevotella*, *Megasphaera*, and *Campylobacter* species than in healthy controls (Dang et al., 2012).

In this study, the Chao1 index and Shannon index of oral microbiomes in both A0 group and B0 group were significantly decreased when compared with HIV-uninfected controls, and the significant difference remained after 12 weeks of ART. We also found that before the ART, the Chao1 index in chronic HIV-infected patients B0 group was significantly lower than that in the acute HIV-infected individuals A0 group, whereas after 12 weeks of the ART, the differences was not significant. Further, after 12 weeks of ART, the Chao1 index in B12 group significantly increased compared with the B0 group. These results suggested that the richness and diversity of oral microbiomes decreased with the progression of HIV infection. Although such reduction can be improved by ART, the oral microbiota dysbiosis cannot be fully restored after the 12-week ART in PLWH (Saxena et al., 2016; Presti et al., 2018). Moreover, there was no significant difference observed in beta diversity when comparing A0 group with B0 group, but a significant difference was noticed between groups A12 and B12. Likewise, no significant difference in beta diversity was found between A0 and A12 groups, whereas there was a significant difference between groups B0 and B12. These results further indicated that the dysbiosis in oral microbiome in people living with acute HIV infection could be partially restored if the ART is early initiated. Recent studies have also revealed that ART initiated in acute HIV infection can limit reservoir size and mitigate systemic chronic immune activation (Cheret et al., 2015; Hey-Cunningham et al., 2015). Altogether, these findings suggested that ART initiated during acute HIV infection might play an important role in protecting commensal bacterial colonization and preventing the occurrence of HIV-related oral diseases.

Alterations in oral microbiota have been noted in individuals with HIV. However, the mechanisms remain unclear and the compromised mucosal immunity might contribute to the dysbiosis of the oral microbiota in PLWH (Heron and Elahi, 2017). It is well documented that Th17 cells are depleted in HIV infection (Cote et al., 2019), however, other studies have shown that oral-resident Th17 cells can play an important role in controlling oral fungal colonization (Conti et al., 2014; Kirchner and LeibundGut-Landmann, 2021). Additionally, salivary components, such as salivary IgA, lactoferrin, defensins, and epithelial cell-mediated cytokines, are altered in PLWH, resulting in the occurrence of frequent oral infections (Muller et al., 1992). A recent study reported that the changes of salivary microbiome in HIV infection and found that *Streptococcus* was enriched in HIV-infected individuals, whereas the richness of *Neisseria* was high in healthy controls (Li et al., 2020). In present study, we observed that the abundance of *unidentified Prevotellaceae* in both groups of patients with acute and chronic HIV infection was increased as well as the abundances of *Prevotella* in acute HIV individuals and *Streptococcus* in chronic HIV individuals.

Interestingly, the changes observed in the oral microbiomes were similar to those in the gut microbiomes in HIV-infected patients. One possible explanation is that oral microbiome could indirectly impact gut bacteria by eating and dispersing (Schmidt et al., 2019). The gut microbes might also in turn affect the oral microbiome through microbial translocation or systemic immune regulation. In addition, it has been shown that *Prevotella* exhibit an increased ability to induce inflammatory mediators, such as IL-6, IL-8, and tumor necrosis factor-α (TNF-α), when compared with strict commensal oral bacteria (Larsen, 2017). The *Prevotella* can also promote periodontitis by driving the recruitment of neutrophil *via* Th17 immune responses (Uriarte et al., 2016). Another study also found that *Streptococcus* was enriched in AIDS patients with periodontitis (Zhang et al., 2015). Moreover, a recent study revealed a significant enrichment of *Streptococcus* in the saliva of HIV-infected individuals with high sCD14 levels, which might contribute to HIV-associated immune activation (Annavajhala et al., 2020). In contrast, several groups of bacteria, such as *Lactobacillus*, *Rothia*, *Lautropia*, and *Bacteroides*, were found in greater abundance in healthy controls. It has been demonstrated that *Lactobacillus* species can produce various antimicrobial factors including hydrogen peroxide, acetic acid, lactic acid, and bacteriocins (Spinler et al., 2008). Salari et al. also demonstrated in an *in vitro* study that the *lactobacilli* have antifungal effects on different oral *Candida* species isolated from HIV/AIDS patients (Salari and Ghasemi Nejad Almani, 2020). It therefore seems that HIV-induced oral microbiota dysbiosis might be characterized by an increased abundance of bacteria that are potentially inflammatory or pathogenic and a decreased abundance of bacteria that are anti-inflammatory or protective.

Although the similar oral microbiome composition has been reported between the HIV-infected individuals undergoing ART and healthy controls, there are significant differences (Li et al., 2020). In the longitudinal study, we collected samples from acute and chronic HIV-infected MSM at baseline and 12 weeks of ART. We found that *Bradyrhizobium* was enriched in both acute and chronic HIV-infected individuals after 12 weeks of ART, whereas *Clostridia*, *Actinobacteria*, *Lactobacillus*, *Ruminococcaceae*, and *Rothia* were enriched in controls. Yang et al. reported that *Bradyrhizobium* was enriched in the proximal gut of PLWH (Yang et al., 2016). They also found that *Lactobacillus* species might have a co-avoidant relationship with *Bradyrhizobium pachyrhizi* in the duodenum. However, little is known about the mechanisms of *Bradyrhizobium* colonization in PLWH, and the exact effects of ART on *Bradyrhizobium* colonization remain to be shown. Furthermore, we found that the abundances of potentially pathogenic bacteria, such as *Prevotella histicola*, *Prevotella melaninogenica*, and *Veillonellaceae*, were decreased when comparing samples collected at baseline with those collected following 12 weeks of ART in PLWH. Our data indicated that ART can partially reverse the effects of HIV on the oral bacteriome, whereas the commensal bacteria with “protective capacity in the oral cavity have been not fully recovered (Moyes et al., 2016).

After 12 weeks of ART, comparing the composition of oral microbiome in people living with acute HIV infection with that of chronic HIV infection, we found that the abundance of *Campylobacter* in the chronic HIV-infected individuals was significantly higher than those in acute HIV-infected individuals. *Campylobacter* spp are gram-negative bacteria with predominant enteric pathogenicity (Manfredi et al., 2002). Molina et al. showed that *Campylobacter* can cause acute diarrhea in PLWH (Molina et al., 1995). In untreated PLWH, *Campylobacter* species in the lingual microbiome was associated with high-level viremia (Dang et al., 2012). However, another study showed that ART increased the risk for recovering *Campylobacter* species in saliva of HIV-positive women (Navazesh et al., 2005).

Of note, we also observed that alterations in the oral microbiome are associated with CD4^+^ T-cell count in patients. The abundances of *Haemophilus*, *Actinomyces*, unidentified *Ruminococcaceae*, and *Rothia* were significantly decreased in subjects with lower CD4^+^ T-cell counts (<200 cells/mm^3^) when comparing with subjects with CD4>200 cells/mm^3^. It is known that *Haemophilus* bacteria can implicate in various opportunistic infections. A previous study reported that *Haemophilus parainfluenzae* was significantly associated with HIV-positive individuals, and it was positively correlated with CD4^+^ T cell counts within the HIV-positive group (Kistler et al., 2015). However, a recent study found that the genus *Haemophilus* was correlated negatively with CD4^+^ T cell count (Li et al., 2020). The possible reasons for the different results might include the different study subjects, samples, the small sample sizes, and other possible factors not involved in the analysis.

One of the limitations of this study was the small sample size. Clearly, studies with large sample size are required. Other factors, such as age, might also affect the results of our study. In addition, previous studies have indicated that oral fungal colonization is altered in HIV-infected individuals (Mukherjee et al., 2014; Hager and Ghannoum, 2018). Although the occurrence of OPC in HIV infection has significantly declined since the introduction of ART, it remains a common opportunistic infection in AIDS diseases (Thompson et al., 2010; Patil et al., 2018). The present study was focused on the oral bacterial community in HIV infection, future studies should also address how the oral mycobiome shifts in the setting of HIV infection and ART initiation.

In conclusion, this longitudinal study has shown important alterations in oral microbiome resulting from HIV infection as well as among MSM with acute and chronic HIV infection before and after ART. The findings might contribute to improved oral health in HIV-infected individuals and provide some clues to exploring the association of the oral and the intestinal microbiome during the disease courses.

## Data Availability Statement

The datasets presented in this study can be found in online repositories. The names of the repository/repositories and accession number(s) can be found below: NCBI SRA PRJNA739016.

## Ethics Statement

The studies involving human participants were reviewed and approved by the Ethics Committee of the Beijing Youan Hospital ([2018]025). The patients/participants provided their written informed consent to participate in this study.

## Author Contributions

All authors made a substantial, direct, and intellectual contribution to the work, including the study design, subject recruitment, sample collection, laboratory experiments, data analysis, and manuscript drafting. All authors contributed to the article and approved the submitted version.

## Funding

This work was supported by the National Natural Science Foundation of China (82072271, 81772165, and 81974303), the National 13^th^ Five-Year Grand Program on Key Infectious Disease Control (2017ZX10202101-004-001, 2017ZX10202102-005-003), Beijing Natural Science Foundation (7172016), the NSFC-NIH Biomedical collaborative research program (81761128001), the Beijing Key Laboratory for HIV/AIDS Research (BZ0089), and Beijing Natural Science Foundation and Handian Innovation Joint Project (19L2043).

## Conflict of Interest

The authors declare that the research was conducted in the absence of any commercial or financial relationships that could be construed as a potential conflict of interest.
