# Supplementary material for: Alteration in Oral Microbiome Among Men Who Have Sex With Men With Acute and Chronic HIV Infection on Antiretroviral Therapy
Source: Front Cell Infect Microbiol. 2021 Jul 14;11:695515. doi: 10.3389/fcimb.2021.695515 (PMC8317457; doi:10.3389/fcimb.2021.695515)

Supplementary Materials

**Supplementary Figure S1.** (A) Rarefaction Curve of the OTUs derived from A0, B0, D, A12 and B12 groups. (B) Rank Abundance of the OTUs derived from A0, B0, D, A12 and B12 groups. A0: people living with acute HIV infection at baseline; B0: people living with chronic HIV infection at baseline; D: HIV-uninfected controls; A12: people living with acute HIV infection after 12 weeks of ART; B12: people living with chronic HIV infection after 12 weeks of ART.


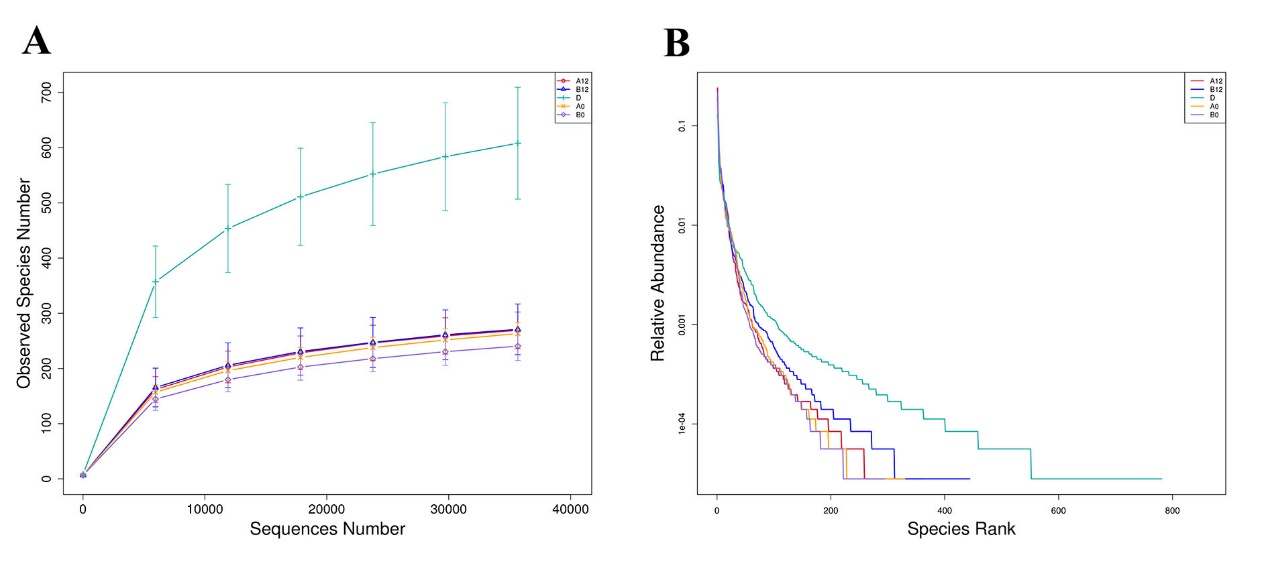


**Supplementary Table S1.**  The Observed-species, Chao1, Shannon, Simpson, ACE, Good-coverage and PD whole tree indices were used to estimate Alpha diversity in A0, B0, D, A12 and B12 groups. A0: people living with acute HIV infection at baseline; B0: people living with chronic HIV infection at baseline; D: HIV-uninfected controls; A12: people living with acute HIV infection after 12 week of ART; B12: people living with chronic HIV infection after 12 weeks of ART.

| **Group** | | **Observed species** | | **shannon** | | **simpson** | | **chao1** | | **ACE** | | **Goods coverage** | | **PD whole**  **tree** | |
| --- | --- | --- | --- | --- | --- | --- | --- | --- | --- | --- | --- | --- | --- | --- | --- |
| A0 | 263 | | 4.337 | | 0.879 | | 322.037 | | 323.432 | | 0.998 | | 20.481 | |  |
| B0 | 240 | | 4.139 | | 0.865 | | 288.990 | | 289.601 | | 0.998 | | 18.243 | |  |
| D | 608 | | 5.820 | | 0.944 | | 725.835 | | 718.575 | | 0.996 | | 41.762 | |  |
| A12 | 269 | | 4.160 | | 0.861 | | 312.213 | | 315.101 | | 0.999 | | 21.353 | |  |
| B12 | 270 | | 4.142 | | 0.855 | | 320.596 | | 316.764 | | 0.998 | | 22.393 | |  |

**Supplementary Figure S2.** Comparisons of the pathways (A) between A0 and D groups. (B) between B0 and D groups. (C) between A12 and D groups. (D) between B12 and D groups. A0: people living with acute HIV infection at baseline; B0: people living with chronic HIV infection at baseline; D: HIV-uninfected controls; A12: people living with acute HIV infection after 12 weeks of ART; B12: people living with chronic HIV infection after 12 weeks of ART.


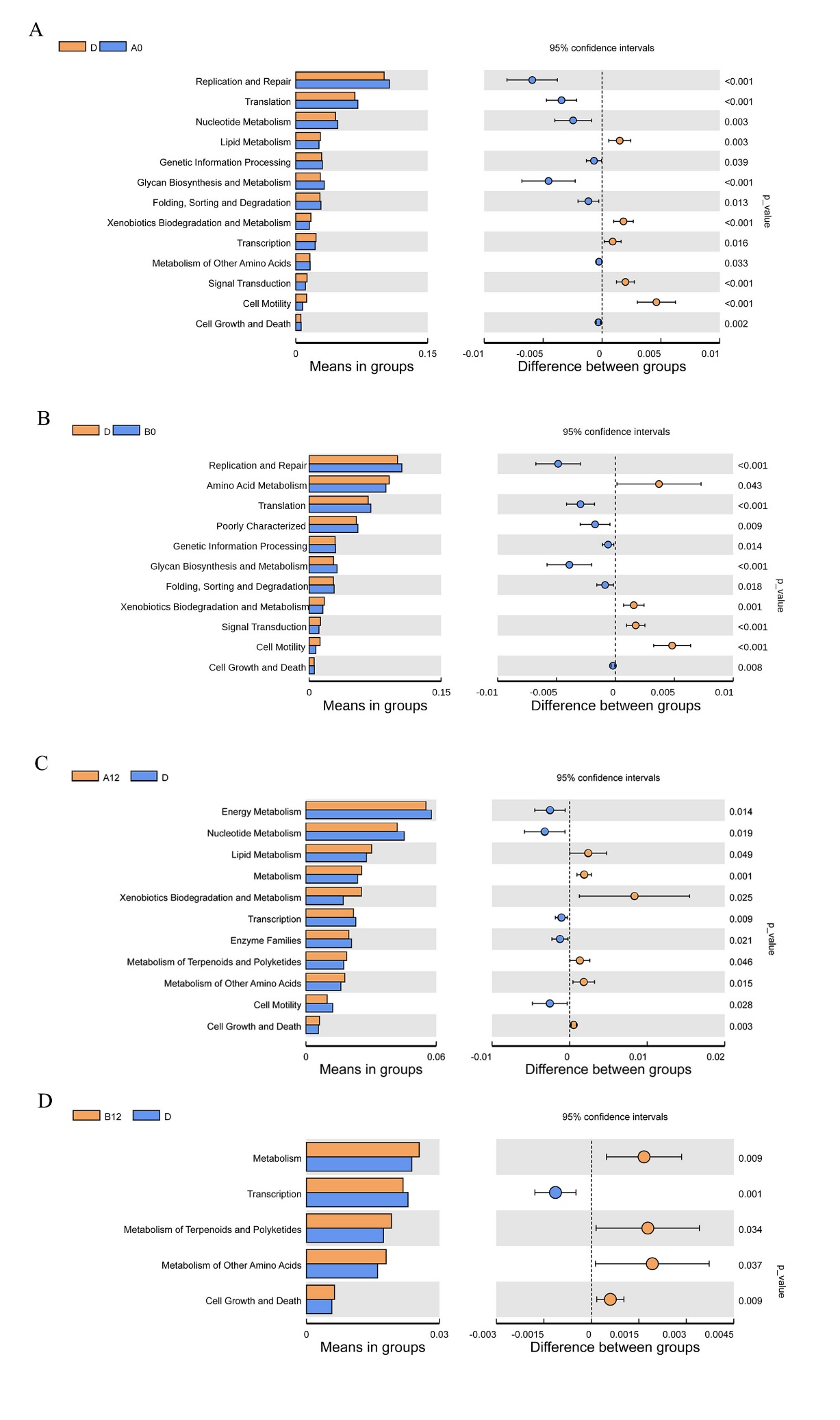

Supplement: Supplementary file 1 [file Table_1.docx]
